# The active surveillance management approach for patients with low risk papillary thyroid microcarcinomas: is China ready?

**DOI:** 10.20892/j.issn.2095-3941.2021.0058

**Published:** 2021-09-24

**Authors:** Wen Liu, Xuejing Yan, Ruochuan Cheng

**Affiliations:** 1 Department of Thyroid Surgery, Clinical Research Center for Thyroid Diseases of Yunnan Province, The First Affiliated Hospital of Kunming Medical University, Kunming 650032, China; 2 Department of Management of Chronic Non-communicable Diseases, Yunnan Center for Disease Control and Prevention, Kunming 650034, China

**Keywords:** Papillary thyroid carcinoma, microcarcinoma, active surveillance, observation

## Abstract

Due to exponential increases in incidences, low risk papillary thyroid microcarcinoma (PTMC) has become a clinical and social issue in recent years. An active surveillance (AS) management approach is an alternative to immediate surgery for patients with low risk PTMC. With decreased doubts about the safety and validity due to evidence from a large number of studies, the AS approach has become increasingly popular worldwide. However, Chinese thyroid surgeons still lag behind other countries in their knowledge of clinical practices and research related to AS. To promote the implementation of AS in China, thyroid surgeons should understand the implications, advantages, and disadvantages of management approaches for AS, and should also consider the willingness of Chinese patients, the impact on the medical billing system, and the enthusiasm of doctors. Thus, a management approach for AS based on the Chinese population should be developed to reduce the risk of disease progression and enhance patient adherence. Herein, we summarize the recent research achievements and deficiencies in AS approaches, and describe the initial experiences regarding AS in the Chinese population, in order to assist Chinese thyroid surgeons in preparing for AS management in the era of PTMC precision medicine.

## Introduction

The global thyroid cancer burden is estimated to have risen to 590,000 new cases in 2020, and thyroid cancer has become the 6th most common malignancy in China, accounting for more than a quarter of the estimated new cases worldwide in 2020^1,2^. The dramatic increase in thyroid cancer is generally attributed to the advance and widespread use of diagnostic technologies such as high resolution ultrasonography. However, some investigators have suggested that for papillary thyroid carcinoma (PTC), there is an epidemic of diagnosis rather than an epidemic of disease^3–5^. From 2002 through 2018, the number of new thyroid cancers rose 11.7-fold (48,621 *vs.* 567,233), but the number of deaths remained largely stable (35,375 *vs.* 41,071)^6,7^. Therefore, over diagnosis and overtreatment of papillary thyroid microcarcinoma (PTMC) have attracted great attention from the medical establishment, and from society as a whole.

Since the 1990s, 2 Japanese centers have conducted prospective observational studies of active surveillance (AS) for patients with T1aN0M0 PTMCs^8,9^. Following exciting outcomes from a series of clinical trials of AS in Japan, clinical practice and research related to AS have expanded worldwide^10,11^. The longer expected monitoring period and the higher lifetime probability of disease progression can be expected, because PTMC occurs at an earlier age comparing with other cancers (e.g., prostate cancer)^12^. However, there are still doubts regarding whether AS should be widely used in China^13^. It is not endorsed by all Chinese surgeons as an alternative to immediate surgery for low risk PTMC patients. To date, there have been no reports on the large-scale use of AS in clinical practice in China. Herein, we summarized research on the use of an AS approach in PTMC management, and described how to implement an AS approach based on the current doctor-patient characteristics and medical policies in China, to assist Chinese thyroid surgeons and patients in transitioning towards the era of PTMC precision medicine as soon as possible.

## The theoretical basis for AS in low risk PTC patients

In autopsy studies, latent, asymptomatic PTMC was present in 4%–11% of autopsy patients who died from nonthyroidal diseases^14^. A systematic review and meta-analysis pooled for over 6 decades by Furuya-Kanamori et al.^14^ confirmed that the average prevalence of occult differentiated thyroid carcinoma was 12.7% in autopsy studies, which examined sectioned whole thyroid glands (5,597 cases)^15^. It was therefore assumed that a certain percentage of PTMC may be resting and inert. Using ultrasound examination and an ultrasound-guided fine needle aspiration biopsy (FNAB), a screening study^16^ reported that thyroid cancer was detected in 3.5% of healthy Japanese women aged ≥ 30 years, with 85% of the thyroid cancers being ≤ 15 mm. This incidence was more than 1,000-fold the prevalence of clinical thyroid cancer in Japanese women during the same period^17^. These results suggested that the incidence of small thyroid cancers was much higher than that of previous cognitive cancers, and that most of them remained stationary and were not life-threatening. Japanese investigators have therefore questioned whether the current treatment decisions are reasonable, and have therefore described an AS approach for patients with low risk PTMC.

However, recent investigations suggested that only approximately half of the increases in PTC incidences could be attributed to enhanced detection or over diagnosis, because of increasing trends observed in larger and advanced PTCs, as well as thyroid cancer mortality^18^. The increase in thyroid cancer mortality was only in patients who were diagnosed with PTC, especially in patients who were diagnosed with advanced PTC^19,20^, even after the treatments were more diverse for these tumors^21,22^. The hypothesis of “over diagnosis” in the prevalence of PTC may therefore be invalid.

Based on these findings, in the context of small and inert tumors involving most cases of PTC, the AS approach should be considered as an appropriate management strategy with epidemiological features.

## Changes in guideline recommendations

Since favorable long-term outcomes for AS in clinical trials performed in Kuma Hospital were published in 2014, an AS management approach to avoid overtreatment has gradually been considered as an alternative to immediate surgery in low risk PTMC patients^23^. In recently updated versions of national/institutional guidelines, AS is recommended as a first-line management approach for patients with low risk PTMC (**Table 1, Supplementary Figure S1**)^10,11,24–36^. However, recommendations in China are inconsistent between health authorities and academic institutions, leading to confusion among surgeons. In addition, the inclusion criteria of the Chinese Association of Thyroid Oncology (CATO) for AS are extremely strict. One study evaluating 2 criteria (Kuma Hospital and the CATO) in 778 patients revealed that many more patients met the Kuma criteria than those who met the CATO criteria (72.6% *vs.* 14.4%)^37^. These strict recommendations may therefore limit the development of an AS approach for low risk PTMC patients in China.

**Table 1:** Updates of the guidelines, and consensus on the recommendation of active surveillance management for low risk papillary thyroid microcarcinomas after active surveillance outcomes from Kuma Hospital were published in 2014^†^

Affiliation	Publication year	Statement type	Active surveillance	Immediate surgery	Reference
BTA	2014	Guideline	○	●	^24^
ATA	2015	Guideline	●	○	^10^
Netherlands	2015	Guideline	○	●	^25^
CATO	2016	Consensus	◑	●	^26^
UK national multidisciplinary	2016	Guideline	○	●	^27^
KTA	2016	Guideline	●	●	^28^
NHCC	2018	Guideline	○	●	^29^
Italian^‡^	2018	Consensus	●	●	^30^
Polish^§^	2018	Guideline	○	●	^31^
JAES	2018	Guideline	●	○	^32^
ESMO	2019	Guideline	●	○	^33^
SEOM	2019	Guideline	○	●	^34^
NCCN	2019	Guideline	●	○	^11^
African	2019	Guideline	●	●	^35^
AAES	2020	Guideline	●	○	^36^

CATO, Chinese Association of Thyroid Oncology; ATA, American Thyroid Association; AAES, American Association of Endocrine Surgeons; BTA, British Thyroid Association; ESMO, European Society for Medical Oncology; JAES, Japan Associations of Endocrine Surgeons; KTA, Korean Thyroid Association; NCCN, National Comprehensive Cancer Network; NHCC, National Health Commission of China; SEOM, Spanish Society for Medical Oncology. ○ Not Recommended; ● recommended; ◑ selective under strict conditions. ^†^For search strategies, see Supplement 1. ^‡^Joint statements of six Italian societies: the Italian Thyroid Association, the Medical Endocrinology Association, the Italian Society of Endocrinology, the Italian Association of Nuclear Medicine and Molecular Imaging, the Italian Society of Unified Endocrine Surgery and the Italian Society of Anatomic Pathology and Diagnostic Cytology. ^§^Joint statements of Polish national societies: the Polish Endocrine Society, Polish Society of Oncology, Polish Thyroid Association, Polish Society of Pathologists, Society of Polish Surgeons, Polish Society of Surgical Oncology, Polish Society of Clinical Oncology, Polish Society of Radiation Oncology, Polish Society of Nuclear Medicine, Polish Society of Pediatric Endocrinology, Polish Society of Pediatric Surgeons, Polish Society of Ultrasonography.

## AS observation cohorts

In recent years, some reports have presented the results of clinical studies on AS in patients with PTC from different countries. We searched the PubMed, Medline, Web of Science, CNKI, and CSCD databases through December 2020, and an additional manual search was undertaken (**Supplementary Figure S2**). Articles not available in English or Chinese were excluded. **Table 2** shows that we identified 7 clinical cohorts of AS in patients with low risk PTC^23,38–44^. No patients died, developed distant metastasis, or experienced other severe adverse outcomes that were attributable to delayed surgery. All clinical studies were prospective observational studies, except for 1 secondary analysis of an observation cohort by Ito et al.^23^ in 2014. However, no randomized controlled trials were identified that compared the outcomes between AS and immediate surgery. More relaxed indications for AS and the timing of delayed surgery were both determined, and the safety of AS was therefore further verified in patients with PTC.

**Table 2:** Clinical studies of active surveillance for patients with low risk papillary thyroid carcinomas

Author, year	Affiliation	Patients	Age(range), year	Tumor diameter (range), mm	Inclusion criteria	F-U protocol	F-U period(range), months	Tumor size enlargement (≥ 3 mm)	Tumor volume increase (> 50%)	Newly developed LNM	Non-progression surgery, cases	FU period after delayed surgery(range), months	Outcomes of delayed surgery
Ito et al.^23^, 2014	Kuma Hospital, Japan	1235	NA	324 tumors ≤ 5, 686 tumors 5–8, 225 tumors 8–10	Diagnosed PTMC by FNAB, cN0M0, without invasion to the RLN or trachea, without high-grade malignancy findings, tumor location not adjacent to the RLN or trachea	US Q 6 to 12 months, FNAB for suspicious lymph node	Mean, 60(18–227)	4.7% in total, 4.9% in 5 years, 8.0% in 10 years	NA	1.5% in total, 1.7% in 5 years, 3.8% in 10 years	NA	Mean, 75(1–246)	1 case residual lobe recurrence
Fukuoka et al.^38^, 2016^†^	Cancer institute Hospital, Japan	384^‡^	Mean, 54.0(23–84)	NA	Diagnosed PTMC by FNAB, cT1aN0M0 (asymptomatic and lymph node < 10 mm)	Clinical exam and US Q 6 to 12 months, chest imaging Q 12–24 months	Mean, 81.6	6.0% in total, 6.3% in 5 years, 7.3% in 10 years	NA	1.0%	NA	NA	NA
Sakai et al.^39^, 2018^†^	Cancer institute Hospital, Japan	61	Mean, 54.4(32–78)	Mean, 11.7(11–16)	Diagnosed PTC by FNAB, cT1bN0M0 (asymptomatic and lymph node < 10 mm), take age, tumour size and other risk factor into account	Clinical exam and US q 6 to 12 months, chest imaging Q 12–24 months	Mean, 94.8(12–204)	6.6% in total, 5% in 5 years^§^12% in 5 years^§^	11.5%	3.3%	5	NA	No differences in surgical procedures, complications, or recurrence compared with immediate surgery^¶^
Tuttle et al.^40^, 2017	Memoria Sloan Kettering Cancer centre, America	291^††^	Median, 51(20–86)	232 tumours ≤ 10, 59 tumours 11–15	Bethesda category V or VI, ≤ 15 mm, without evidence of ETE, cN0M0, TSH level within the reference range	US Q 6 to 12 months for 2 years, then annually	Median, 25(6–166)	3.8% in total,2.5% in 2 years,12.1% in 5 years	12.4% in total, 11.5% in 2 years24.8% in 5 years	0%	5	Mean, 7.3(3–32)	No biochemical or structural persistence or recurrence
Oh et al.^41^, 2018	Multicenter^‡‡^, South Korea	370	Mean, 51.1	Mean, 5.9	Diagnosed PTMC by FNAB or CNB, without aggressive variant subtype, without evidence of ETE, cN0M0, tumour location not adjacent to the RLN	Clinical exam and US Q 6 to 12 months, FNAB and thyroglobulin washout for suspicious lymph node	Median, 32.5 (IQR21.5–47.6)	3.5% in total, 0.6% in 2 years, 6.4% in 5 years, 12.0% in 6 years	23.2% in total, 6.9% in 2 years, 36.2% in 5 years, 47.5% in 6 years	1.4%	28	Median, 18.7(IQR7.7–32.2)	No recurrence
Sanabria^42^, 2018	Medellin, Colombia	57	Mean, 51.9(24–85)	Mean, 9.7	Bethesda category V or VI, < 15 mm, encapsulated, cN0	US, the interval not disclosed	Median, 13.3(0–54)	3.5%	NA	0%	2	NA	NA
Rosario et al.^43^, 2019	Belo Horizonte, Brazil	77	Mean, 52(23–81)	69 tumors ≤ 10, 8 tumors 11–12	≥ 20 years, Bethesda category V or VI, ≤ 12 mm, without evidence of ETE, cN0,	US Q 6 months, maintain TSH between 0.5–2.0 mIU/L in patients with TSH > 2.5 mIU/L	Mean, 24.4(6–42)^§§^	1 case suspicious capsular invasion (after F-U 30 months)	NA	0%	2	NA	NA
Molinaro et al.^44^, 2020	University Hospital of Pisa, Italy	93	Mean, 44	Mean, 9.4	≥ 18 years, Bethesda category V or VI, ≤ 13 mm, single malignant (or suspicious) nodule, without evidence of ETE, cN0M0, without hyperthyroidism or concomitant antithyroid drugs, without previous history of thyroid surgery	US Q 6 to 12 months for 2 years, then annually	Mean, 19(6–54)	2.2%	16%	1.1%	19	Median, 18(6–36)	Excellent response

CNB, core needle biopsy; ETE, extrathyroidal extension; FNAB, fine-needle aspiration biopsy; F-U, follow-up; IQR, interquartile range; LNM, lymph node metastases; NA, not available; Q, quaque (every); RLN, recurrent laryngeal nerve; TSH, thyrotropin; US, ultrasonography. ^†^Both studies were from the cohort of the Cancer Institute Hospital, Japan. ^‡^Included 480 lesions from 384 patients with PTMC.^ §^Overall disease progression rate at 5 years and 10 years, including tumor enlargement > 3 mm and new LNM appearance. ^¶^All of < 15 mm cases were not recurrence. ^††^Included 5 patients who was inappropriate for observation (3 for suspicious minor ETE into overlying strap muscles and 2 for suspicious subcentimeter LNM), afterwards, 2 patients were treated with delayed surgery for an increase in tumor size and volume after 12 and 18 months of AS, respectively. ^‡‡^Asan Medical Center, Samsung Medical Center, and Seoul St. Mary’s Hospital. ^§§^Inference was based on the number of follow-up visits for cases.

The favorable outcomes of these studies have greatly increased confidence in investigators, which showed that percentages of tumor growth and the appearance of new lymph node metastases (LNMs) were extremely low, no fatal prognostic impact or life-threatening recurrence occurred from tumor growth or newly developed LNM in patients with delayed surgery, and no patients died or developed distant metastases during the AS period^23,45,46^. Because of the difficulty of randomized controlled trials, long-term observational results from Japanese cohorts might be the best clinical evidence available. Along with promotion and popularization, AS has become the preferred choice among 53.8% of adult patients with low risk PTMC, according to a 2018 survey conducted by members of the Japanese Endocrine Surgery Association (JAES)^47^ (**Table 3** shows that patient characteristics were not appropriate for AS according to JAES standards^48^).

**Table 3:** Indications of inactive surveillance from the Japanese Endocrine Surgery Association consensus statements for papillary thyroid microcarcinomas^48^

Indication of non-AS	
1.	Presence of clinical lymph node metastasis or distant metastasis (rare)
2.	Clinically apparent invasion into the RLN or trachea
3.	Diagnosis of aggressive subtype of papillary thyroid carcinoma on cytology (rare)
4.	Tumors adherent to the trachea, possibly invading
5.	Tumors located along the course of the RLN
6.	Associated with other thyroid or parathyroid disease requiring surgery
7.	Age < 20 years (no current evidence)

AS, Active surveillance; JAES, Japan Associations of Endocrine Surgeons; RLN, Recurrent laryngeal nerve.

## Indications for AS in low risk PTC

The ideal indications for AS should consider 2 principles: the possibility of tumor progression and the possibility of a decline in the expected prognosis after tumor progression occurred. Referring to evidence of previous studies on the clinicopathological characteristics of PTC, clinical centers have established indications for both AS and immediate surgery, based on the above 2 principles.

All tumor progression is theoretically due to continuous mitosis and cell proliferation. Larger tumors are therefore more likely to exhibit continuous growth in size before they are detected. The indications of AS for PTC were expanded to 15 mm, which also led to satisfactory clinical outcomes in several centers^39,40,42^. Nonetheless, some concerns were reported in these cohorts when compared with 2 AS cohorts from Japan (means of 60 and 81.6 months for the follow-up)^23,38^. AS was performed for a shorter duration (median: 25 months) in a cohort studied by Tuttle et al.^40^, in which a higher percentage of tumor enlargement was found during the follow-up. Expanding AS indications therefore cannot be supported, because there is still a scarcity of long-term follow-up evidence, which may increase the risk of progression and may not be conducive to the worldwide promotion of the AS approach.

**Figure 1:**
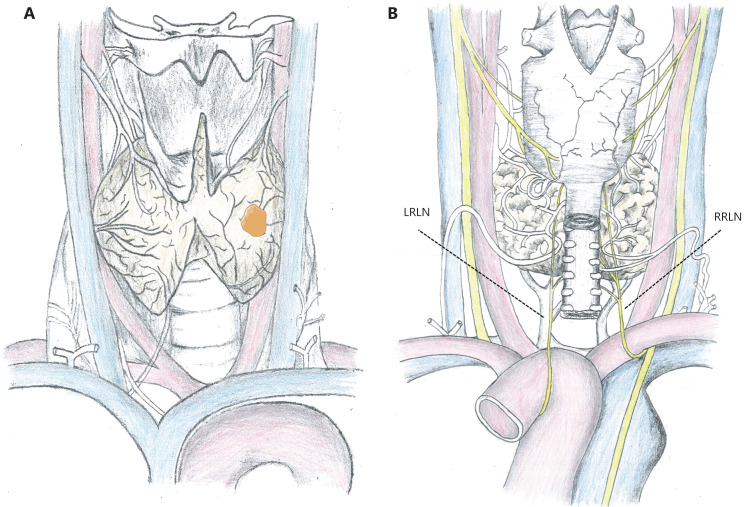
Anatomical position diagram of the thyroid. (A) Anterior view. (B) Posterior view. LRLN, left recurrent laryngeal nerve; RRLN, right recurrent laryngeal nerve.

**Figure 2:**
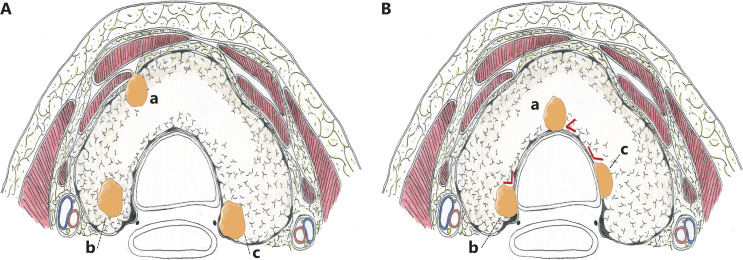
Axial plane diagram of the thyroid. (A) The different levels of risk for the presence and absence of a normal rim between tumor and adjacent tissues. (a) The strap muscles are infiltrated when tumors located on the ventral side progress and penetrate into the thyroid capsule. (b) Tumors located on the dorsal side with a normal rim between the tumor and the course of RLN. (c) Tumors located on the dorsal side without a normal rim between the tumor and the course of the RLN. RLN, recurrent laryngeal nerve. (B) Different angles between the tumor surface and tracheal cartilage. (a) Acute angle; (b) Nearly right angle; (c) Obtuse angle.

Tumors 2–3 mm away from the surrounding thyroid capsule are optimal candidates for AS, because they have dedicated space for potential tumor growth, although it is not an easily accessible condition in patients with small thyroids. In general, tumor enlargement can be detected in a timely manner during an ultrasonographic monitoring interval of 6 months, while it is still in the capsule of the thyroid (**Figure 1**). The consequences of tumor enlargement may vary widely by location. For example, for tumors on the ventral side of the thyroid, only additional partial resection of the strap muscles is required, which minimally impacts the patient’s quality of life (QoL), even if the tumor progresses and penetrates into the thyroid capsule (**Figure 2A**). In addition, tumors located adjacent to the lateral capsule of the thyroid rarely invade the carotid artery. However, tumors located on the dorsal side or paratracheal areas may result in intractable recurrent laryngeal nerve (RLN) palsy or tracheal infiltration once the capsule is invaded, and complications may be unavoidable (**Figures 1B and 2**). Ito et al.^49^ analyzed the tumor location features of 1,143 patients with low risk PTMC who underwent immediate surgery, and found no invasion into the tracheal or RLN in tumors < 7 mm. In PTMCs > 7 mm, which were attached to the trachea with an obtuse angle, 12 (24%) of 51 tumors required cartilage shaving or resection of full layers of the trachea. However, in tumors and tracheas with a nearly right/unclear angle or acute angle, no shaving or resection of the trachea was performed. The contact surface was greatest when the PTMC was at an obtuse angle to the tracheal wall, so a higher risk of infiltration was possible (**Figure 2B**). However, angular measurements have been based on previously stored images, using ultrasonography and plain computed tomography (CT) scans, which may cause selection bias. For example, different levels may be measured at different angles in CT images of irregular lesions. Similarly, 9 (9%) of 98 PTMCs ≥ 7 mm without a normal rim between the tumor and the course of the RLN required shave resection, partial layer resection, or resection with reconstruction of the RLN during surgery, whereas there was no clinically important invasion of the RLN in 28 patients with a normal rim (**Figure 2A**)^49^. Therefore, in addition to tumor size, ultrasonic measurements should also include the distance between the tumor and the closest adjacent organ or capsule during AS. Based on current limited evidence, patients with PTMC in suitable locations should be candidates for AS, which could avoid affecting the prognoses and reducing the QoL of these patients if tumor progression occurs.

## Reasonable monitoring indices and proper operation timing

### Evaluation for the enlargement of tumor size

In 2007, enlargement was defined by Ito et al.^50^ as an “increase in maximal diameter of ≥ 3 mm” during AS. This definition is simple and the size is easy to measure, and there have been no reports of RLN paralysis or distant metastasis in long-term observation cohorts in Japan^45,51^. This approach is subsequently being implemented in many centers. Recently, centers from the Republic of Korea and the United States, using 3-dimensional measurements, which can detect changes in tumor size earlier and more accurately, suggested a 50% increase in tumor volume (length × width × height × π/6)^40,52^. Tumor growth kinetic assessment showed a typical linear increase in tumor volumes of ≥ 50%, with a median doubling time of 2.2 years (range: 0.5–4.8 years)^40^, which might be used as a potential prognostic indicator for tumor growth. However, the sensitivity of this indicator is much higher than an increase in diameter of 3 mm. Considering the experiences of the Japan Center, a ≥ 50% increase in tumor volume may be too radical as an independent indicator for progression. Conversely, if tumor volume increases of > 50% are not detected within 2–3 years of close monitoring (twice a year), this can be used as an indicator to extend the intervals between examinations. Further research is expected to verify these findings. In addition, ultrasound measurement is based on sagittal and transverse section images, because it is difficult to ensure precision when 3 dimensions are measured. In addition, measurement of tumor volume is subject to the potential subjective influence of sonographers. Therefore, an increase in maximal tumor diameter of ≥ 3 mm should be considered when evaluating tumor enlargements.

### Evaluation of the appearance of new LNM

Two Japanese studies (mean follow-ups of 60 and 81.6 months) showed that the percentages of newly developed LNM were as low as 1.5% and 1.0%, respectively^23,38^. However, another study by the same authors confirmed that the LNM percentage was as high as 50.5% in 594 PTMC patients who underwent lymph node dissections^8^. Thus, LNM can be underestimated when ultrasonographic evaluation is used, regardless of whether it is used at the beginning of AS or during AS, which is also a main concern of Chinese investigators who oppose AS management for PTMC. In addition, the low sensitivity of ultrasonography is mainly reflected in small cervical lymph nodes rather than in large nodes, which can usually be evaluated with satisfactory accuracy by ultrasound and FNAB. According to the 2015 American Thyroid Association (ATA) guidelines, the prognoses of patients who have ≤ 5 pathological N1 metastases of a small size are not as poor as those of patients who have clinically apparent metastases (clinical or radiological N1 metastases)^53,54^. A study of 2,329 patients with cN0 PTMC showed that the number of LNMs was more than 5 in only 94 cases (4.0%), and male sex and ages < 40 years were independent risk factors [odds ratio (OR): 5.79; 95% confidence interval (CI): 3.67–9.10; *P* < 0.001 and OR: 2.69; 95% CI: 1.64–4.32; *P* < 0.001, respectively]^55^. Although the percentage of LNM with a poor prognosis (> 5 pathological N1) is relatively low in low risk PTMC patients, prediction models that include a larger sample and more variables are still necessary.

### Appropriate operation timing

Tumor enlargement ≥ 3 mm has not been considered the cut-off for surgical intervention in the JAES consensus statement for AS^48^. Miyauchi and Ito suggested that AS could continue until the tumor diameter reached 13 mm if the patients preferred this approach^56^. This is mainly because a 3 mm increase in patients with PTMC who undergo AS, with the upper limit of tumor diameter being 10 mm, results in a 13 mm tumor. Another study reported similar outcomes between T1b and T1a tumors during AS^39^. A study including 824 low risk PTMC patients showed that the growth rate of the tumor decreased significantly after its enlargement. Among tumors with ≥ 3 mm enlargement and an increase in volume ≥ 50%, only 7.7% and 3.8% had continuous rapid increases (tumor doubling rates > 0.5/year), respectively; 12.8% and 12.1% slowly increased (tumor doubling rates: 0.1–0.5/year), respectively, and the rest remained stable or shrunk^57^. Surgical intervention is therefore not the only option, even if tumor enlargement is ≥ 3 mm or has a diameter > 10 mm. Appropriate surgical timing should therefore be comprehensively decided based on tumor location, continuous growth rate, and individual patient preferences.

## Factors affecting decision making in AS

### Age

The only general consensus predictor of tumor growth (≥ 3 mm) or LNM appearance is age. According to a report from the Kuma Hospital, the 10 year progression (tumor enlargement ≥ 12 mm or the appearance of LNM) was 2.5% in patients aged ≥ 60 years, 4.9% in patients aged 40–59 years, and 22.5% in patients aged < 40 years. Younger age significantly predicted PTMC progression outcomes using multivariate analysis (OR: 4.348; 95% CI: 2.293–8.196; *P* < 0.001)^23^. As patient age increased, the estimated lifetime probability of tumor progression significantly decreased from 48.6% in patients aged 20–29 years to 3.5% in patients aged 70–79 years during AS^58^. Nonetheless, younger patients should not be excluded from AS, although they might have a higher risk of tumor progression. The lifetime probability of progression is less than 50%, even in patients aged 20–29 years, and delayed surgery still has an excellent outcome even if tumor progression occurs during AS. The duration of AS from initiation to progression could therefore be regarded as the “bonus” period of AS, with no risk of complications and no need for additional thyroid hormone supplementation. Moreover, Ito et al.^59^ reported a similar favorable prognosis during AS in 50 gestational women with a low risk of PTMC. The prevalence of PTMC is much higher in women than in men. Young women are more likely to desire pregnancy than women of other ages. Therefore, AS can significantly reduce the dual requirements of surgery and pregnancy for thyroid hormone medication adjustment, when compared to immediate surgery.

### Multiplicity

Ito et al.^23^ reported that multiplicity was not a risk factor for tumor enlargement of ≥ 3 mm or the appearance of LNM during AS. In another study that investigated AS for low risk PTMC patients, the 10 year progressions of multiple (115 cases) and solitary foci (456 cases) of PTMC patients were 14.8% and 12.2%, respectively (*P* = 0.51)^60^. A classic case in our center was used to support that multiplicity is not associated with disease progression. A patient with 2 PTC foci presented with different biological processes. One process increased from 10 mm to 14 mm (observed for 23 months), while the other remained stable (4 mm, observed for 49 months). The incidence of multifocal cancer (33.5%), especially bilateral cancer (26.6%), is higher in China than in Western countries^61^. Total thyroidectomy is thus recommended as the initial treatment in multifocal PTMC patients who prefer immediate surgery, of which the risk of complications consequently increases. Multifocal PTMC may therefore be more suitable for AS management, as long as the cancer is not omitted and well documented during AS.

### PTMC characteristics using ultrasonography

The calcification features using ultrasound were associated with PTMC progression during AS in the study, which followed 484 cancer foci for a mean period of 6.8 years, and were also associated with patient ages. Fokuoka et al.^38^ found that tumors with coarse calcification and rim calcification had a lower incidence of enlargement than tumors with microcalcification and without calcification. Another study, also from Japan, confirmed that in 180 patients who underwent delayed surgery after 1 year of AS, there was no macrocalcification ≥ 2 mm with acoustic shadow in 18 of all enlargement tumors^62^. In contrast, Oh et al.^63^ reported that a tumor volume increase of ≥ 50% more commonly occurred in PTMC with macrocalcification. One study evaluated the relationship between tumor blood supply and progression, and showed that the likelihood of 3-mm diameter growth in 70 lesions with a rich blood supply at the initiation of AS was significantly higher than that in 410 lesions with a poor blood supply (14.6% *vs.* 4.6%). It is worth noting that 61.4% of patients with PTMC showing rich vascularity at initial assessment had decreased vascular density during AS^38^. In addition, the rich blood supply in PTC was not related to LNM, as reported in our previous study^64^.

The characteristics of tumors are not invariable, and “risk factors” at the initiation of AS might change with prolonging of the observation duration. Therefore, there is limited evidence that calcification type, blood supply distribution, and other ultrasonographic features could not be used as indicators to evaluate the feasibility of AS.

## Other controversies related to AS

### Is FNAB necessary?

In earlier published decision-making, FNAB was not considered an absolute inclusion criterion for AS by the Memorial Sloan Kettering Cancer Center^65^. However, a subsequent study of 291 patients reported that the inclusion criteria of AS were still defined as Bethesda category VI or V with suspicious ultrasonographic features^40^. Other study cohorts have also agreed that FNAB should be necessary (**Table 2**).

There are several advantages to FNAB before AS. First, the diagnosis of malignancy can be further confirmed. A multicenter study reported an accuracy ranging from 77.4%–82.8% when evaluating suspected malignant nodules by different ultrasonic grading systems^66^. The ultrasonographic evaluation interval was shortened from 2 years to 6 months, when these tumors were misdiagnosed as malignant tumors. However, the diagnostic performance of ultrasonographic evaluation was improved by using experienced sonographers or with the popularization of artificial intelligence in the diagnoses of thyroid nodules^67,68^. Second, although they might avoid delayed treatment due to AS, microanaplastic carcinomas and aggressive subtypes of PTC can only be rarely identified by FNAB. One patient still had a tall-cell variant of PTC (Bethesda V at the initiation evaluation of the AS), with newly appeared LNM after 12 months of follow-up in a FNAB filtered cohort in Italy^44^. In a Republic of Korea cohort, 3 patients underwent delayed surgery and were histopathologically diagnosed with tall-cell variants^41^. FNAB is not considered a conventional examination for nodules < 1 cm in the 2015 ATA guideline update, which may be due to a comprehensive trade-off for both low incidence and low detection performance for aggressive thyroid cancers^10^.

The disadvantage of FNAB is that it may decrease acceptance of the AS approach for patients. Patients with “confirmed PTMC” may feel increased anxiety compared to those with “suspected PTMC.” In patients who do not undergo FNAB during AS, the overall risk of disease progression is not increased; the cost of ultrasonic monitoring is increased, but the workload of doctors may also increase. We therefore recommend that in China, continuous ultrasonic monitoring may be superior to FNAB during AS for nodules in ideal locations. Additionally, despite the cumulative incidence of needle tract implantation being extremely low (0.37% and 0.58% from thyroid tumors and lymph nodes at 10 years after FNAB, respectively), tract implantation was associated with possible histological transformation for more aggressive subtypes than the original counterparts^69^.

### Should TSH suppression therapy be performed?

Sugitani et al.^70^ reported that thyrotropin (TSH) levels at diagnosis were not considered an independent factor for tumor enlargement, based on the mean follow-up of 6.5 years from 323 patients. Another study from the Republic of Korea^71^ reported that high TSH levels (median: 3.11 mU/L) were independently associated with tumor progression. The study also predicted that a TSH level of 2.50 mU/L was an ideal cut-off of PTMC progression based on the K-M curve. TSH is considered to be a growth factor in thyroid follicular cells that affects the occurrence and progression of follicle-derived thyroid cancer. It regulates thyroid cell differentiation by modulating the expression of genes that play an important role in transcription of the sodium-iodine symporter gene, which is significantly downregulated after prolonged TSH suppression, resulting in reduced tumor progression. However, active TSH suppression may increase adverse effects of the heart or bone in specific populations, such as older patients or perimenopausal women. Active TSH suppression has therefore not been proven to be beneficial in preventing tumor progression during AS for low risk PTMC patients. However, for patients with high basal TSH levels or young patients, the benefits of maintaining TSH at the low level of the normal range may outweigh the risks.

### Patient preference

Patients depend heavily on the professional knowledge and skills of medical personnel, but no medical or research purpose should take precedence over the rights and interests of these patients. Hence, it should be fully respected that patients can change their preference for surgery during AS. Regarding the anxiety of PTMC patients, both the AS management approach and immediate surgery complement each other. Patients who undergo AS can avoid invasive surgery and its complications, so anxiety is largely related to disease progression. In contrast, anxiety in patients who undergo surgery may persist over time and be related to surgical complications and cancer recurrences, rather than disease progression. In a study of 234 patients under AS by Davies et al.^72^, 37% were anxious about cancer progression, and 60% of the patients reported less anxiety during AS than when the cancer was found. Furthermore, AS was considered the most suitable management approach by 83% of the patients. Other studies also showed that the mental health of the AS group was better than that of the surgery group^73,74^.

## How can AS implementation be facilitated in China?

Since 2015, ATA guidelines have recommended that an active surveillance management approach be considered as an alternative to immediate surgery, which has been controversial^10^. Chinese thyroid surgeons, including those at our center, do not endorse the AS approach in managing patients with low risk PTMC because of the following concerns: (i) Chinese surgeons and practical guidelines prefer prophylactic lymph node dissection as the initial treatment for PTMC patients, who usually have a high percentage of pathological LNM. Many patients may already have lymph node micrometastases at the initiation of AS. (ii) The traditional philosophy of Chinese patients is not consistent with the notion of “living with a tumor.” (iii) There is a serious imbalance in the doctor-patient ratio in China; it is therefore difficult for surgeons to achieve close monitoring and active follow-up for PTMC patients who undergo AS. (iv) Due to the convenience of the clinical consultation environment, hospital visits are often not established with Chinese patients, which increases the difficulty of follow-ups during AS.

With an increased understanding of the AS approach, our center has been conducting a prospective observational study on the AS management approach for low risk PTMC Chinese patients (ChiCTR2000032623). We analyzed the differences in cancer burden, patient characteristics, surgeon concerns, medical care systems, and adherence between China and other countries based on our own experiences with AS management in the Chinese population, to implement an AS approach in patients with low risk PTMC in China.

### The significance of AS management and the burden of thyroid cancer

A total of 21% of cancer patients met the WHO standard of poverty due to illness (healthcare costs were > 30% of household income) in China^75^. If a disease with an excellent prognosis requires large amounts of financial resources due to over diagnosis and overtreatment, it is conceivable that other diseases will not receive enough investment by the government. According to estimates in the 26 major countries analyzed, over 830,000 thyroid cancers in female patients might have been over diagnosed between 2008 and 2012. Among these patients, approximately 390,000 were in China, and the estimated percentage of thyroid cancer in women attributable to over diagnosis was approximately 87%, which ranked third in the world^76^. The age-standardized incidence of thyroid cancer in China is much higher than the world average [11.3/10^5^ *vs.* 6.6/10^5^ (2020)]^2^; moreover, there is an obvious difference in geographical distribution, with the annual number of thyroid cancers in the eastern, more developed area being approximately 4.4-fold higher than that in the western area^77^. If this continues, the overtreatment and burden of thyroid cancer may further increase, along with China’s economic development.

Studies from Australia and Hong Kong (China) showed that the medical costs for immediate surgery were equivalent for AS for approximately 16 years^78,79^. In Japan, immediate surgery costs were 4.1-fold greater than AS costs over 10 years^80^. Insurance policies and medical costs vary in different countries, but the differences between the costs of surgery and AS may be very obvious due to the extremely low cost of outpatient services in China. In addition, the surgical composition ratio for PTMC at our center (approximately 70%) showed a consistent trend with the increasing incidence of PTMC. There were 2,659 (47.9%) patients with cN0 stage PTC who could be considered potential candidates for AS between July 2016 and June 2020. These cases resulted a hospitalization costs of 38,034,347 Yuan (approximately 5.8 million dollars); however, the indirect cost caused by missed time at work, the use of transportation, etc., could not be estimated. Moreover, thousands of surgeries resulted in thousands of patients who needed to undergo additional thyroxine supplementation. Unnecessary surgery and related complications may increase the physical and psychological burdens of patients with PTMC. Therefore, use of the AS approach for low risk PTMC patients in China should not be delayed.

### Patient characteristics and surgeon concerns

In our observation cohort, all 82 patients were detected by ultrasound health examination, and 57.1% (54/98) of tumors were < 5 mm in size. A common feature of patients in our cohort was that they usually had urban employee medical insurance (UEMI) and underwent thyroid ultrasound examination every 1–2 years. The odds of regular health checks among patients with UEMI was 52% higher than those with rural/urban resident medical insurance, because employers often offer health checks as incentives^81^. Smaller PTCs are more frequently detected in patients who are also involved in the over diagnosis of thyroid cancer in China. Three patients underwent tumor progression (2 cases of enlargement > 3 mm and 1 case of suspicious capsular invasion) with initial sizes of 5, 6, and 10 mm. In addition, a growing body of high quality data indicates that patient outcomes are not significantly affected by prophylactic lymph node dissection^52,82^. The majority of lymph node micrometastases do not develop into clinically apparent metastases, especially in patients with smaller PTC sizes. There may be an erroneous fear by Chinese surgeons of “a high rate of LNM in China.”

Another feature of patients with UEMI is that they are usually well-educated, and more easily understand the concept of precision medicine and prefer to be treated by AS. One patient underwent delayed surgery due to a preference change rather than disease progression during the median AS 9 (interquartile range: 4–18 months). Based on our limited experience, Chinese patients have a lower percentage of non-progression surgery than foreign cohorts during AS, which was inconsistent with the perceptions of Chinese scholars that patients could not accept “living with a tumor.”

### Enhancing adherence during the follow-up

If an authoritative thyroid surgeon recommends AS and tailors the AS strategy for patients with low risk PTMC, patient anxieties will be reduced, and confidence in the AS approach will be increased. If 1 clinician is well-informed of AS management practices and provides reasonable communication and follow-up to patients, it may increase patient adherence. At the initiation of AS, 1-on-1 international clinical practice status of AS introduction, and advantages and disadvantages of AS management analysis were conducted by clinicians. Patients who were diagnosed using cytopathology or ultrasonography were highly accepting of the AS approach (only 2 candidates rejected AS). In addition, the clinician is responsible for communication and consultation with patients through instant messaging applications during AS, which can greatly relieve the patient’s anxiety.

A recent study showed that proceeding to AS without FNAB is a safe procedure in selected patients, and FNAB could be postponed until disease progression^83^. An increased frequency of ultrasound re-examination may be more acceptable to Chinese patients than invasive procedures, because it is inexpensive and convenient. Two options involving FNAB or the multiple ultrasound + calcitonin test were offered to patients, who have similar diagnostic efficiencies for excluding medullary carcinoma, which were minimally effective for anaplastic carcinomas and aggressive subtypes of PTC. FNAB was chosen in approximately 30% of patients in our cohort, and 1 patient was cytologically diagnosed as benign after 1 year of AS. To date, this protocol has not resulted in a poor prognosis due to delayed diagnosis during AS.

### Reform of the medical billing system

The implementation of the AS approach is essential both in the individual treatment of patients and in reducing burdens on the country. Both in developed and some developing countries, the AS approach has resulted in favorable outcomes. In contrast, although China’s economic level and the average level of education are higher, implementation of AS is expanding slowly. This may be related to the medical billing system of China. Primary medical costs are concentrated in medical procedures (e.g., surgery) and equipment usage (e.g., laboratory and imaging examinations). Conversely, the process of disease diagnosis through professional knowledge is extremely inexpensive, although it usually requires a great repository of knowledge and experience by clinicians. The AS approach is a reflection of the need for individualized diagnosis and treatment for PTMC, which necessitates long-term or even lifelong meticulous and comprehensive evaluations. Follow-up and surveillance are mostly performed by young residents or attending physicians (level 1 or 2 in the 4 level physician hierarchy in China). However, the medical billing value for AS management cannot match the value for physician labor (usually less than $2 per outpatient visit). To promote AS management for PTMC in China, it may be necessary to reform the medical billing system for diagnosis and treatment, to fully motivate both clinicians and medical institutions to embrace this approach.

### Visions for future research

The use of AS in the precision treatment of thyroid cancer is a landmark development. With the promotion of AS management worldwide, it is expected that the current status of overtreatment for PTMC will be significantly improved in the future. Several specific molecular signatures were found to be associated with more aggressive behavior in PTC (e.g., BRAF or BRAF+TERT promoter)^84,85^. However, although clinical observational research for AS is rapidly progressing, molecular markers that can accurately predict the biological behavior of PTMC have not yet been identified. Yabuta et al.^86^ at Kuma Hospital divided 26 patients who underwent delayed surgery during AS into 3 groups: disease stable, tumor enlargement ≥ 4 mm, and newly developed LNM. There was no significant difference in BRAFV600E mutation status (64%, 70%, and 80%, respectively), and no TERT promoter mutation was found in any patient. Another study reported that the Ki-67 labeling index was associated with PTMC enlargement during AS, and was usually detected using postoperative histopathological specimens^62^. Theoretically, different biological behaviors may be driven by various genetic events in the same histological type of tumor. Although mutation-prone gene loci have been identified in large samples of PTC by the Human Genome Project, these findings may instead be a constraint on the direction of AS^87,88^. Whether inert or progressive tumors are confirmed in clinical samples after long-term observation is unknown. Whether enough such samples were included in the testing samples of the Human Genome Project is still in doubt, and whether the gene testing panel, which is presently widely used in Chinese clinical practice, covers all valuable molecular markers is also unknown. A recent study^89^ reported a synthetic lethal partner of BRAF^V600E^ in thyroid cancer, which motivated us to ask the following question: is PTC progression based on synergy between BRAF and other gene mutations? It also suggests that there is much work left to do in characterizing the biological behavior of low risk PTC. There are many constraints, such as a long follow-up period, few surgical cases, and difficulty in collecting tumor specimens during the AS management process. Clinical research and basic research complement each other, and research on the PTMC progression mechanism must be based on a larger number of AS cases. Chinese thyroid surgeons should therefore include more low risk PTMC patients who meet AS criteria in the observation cohort.

## Conclusions

In summary, with doubts regarding the safety and validity of AS decreasing due to evidence from a large number of studies, this approach has been increasingly popularized worldwide. As a thyroid surgeon, it is my honor to witness the course of low risk PTC being developed by Japanese teams led by Professor Miyauchi. Their bold hypotheses, careful verification of scientific research, and persistent pursuit of scientific truth are admirable. Chinese thyroid surgical teams have lagged behind in clinical practice and research related to AS, but there are still many unsolved questions to be answered in regard to the management approach and mechanism of AS, especially involving biomarkers of tumor progression in the Chinese population, which should be given high priority. China has the inherent advantage of a large number of cases, and we sincerely hope that thyroid surgical teams in China can join in the implementation of the AS approach for patients with low risk PTMC as soon as possible, so that they can cooperate with each other to conduct multicenter and related basic research.
