# Supplementary material for: The active surveillance management approach for patients with low risk papillary thyroid microcarcinomas: is China ready?
Source: Cancer Biol Med. 2021 Sep 24;19(5):619–34. doi: 10.20892/j.issn.2095-3941.2021.0058 (PMC9196058; doi:10.20892/j.issn.2095-3941.2021.0058)
Supplement: Supplementary file 1 [file cbm-19-619-s001.pdf]

Supplementary materials

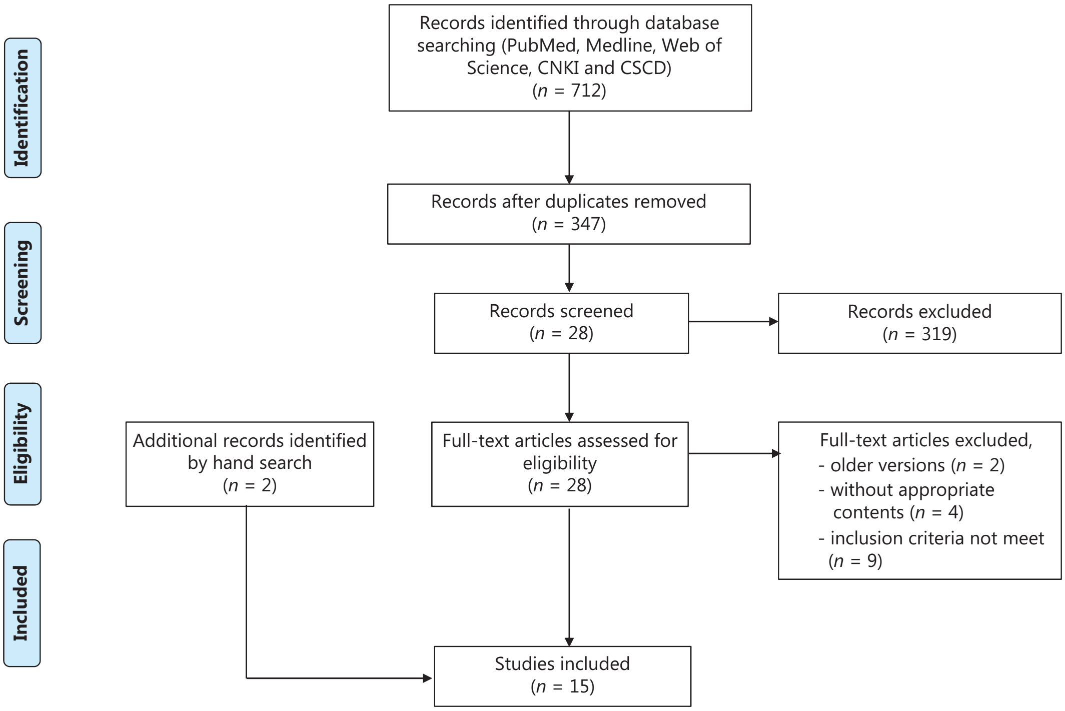

Figure S1 PRISMA flow chart of the included guidelines/consensus. The medical subject heading searches used all combinations of the following index terms: thyroid neoplasms, guidelines, and consensus. Articles not available in English or Chinese editions were excluded. Guidelines regarding other types of histology/diseases, other populations (e.g., pediatric and pregnant populations), diagnosis, or RAIA alone were excluded.

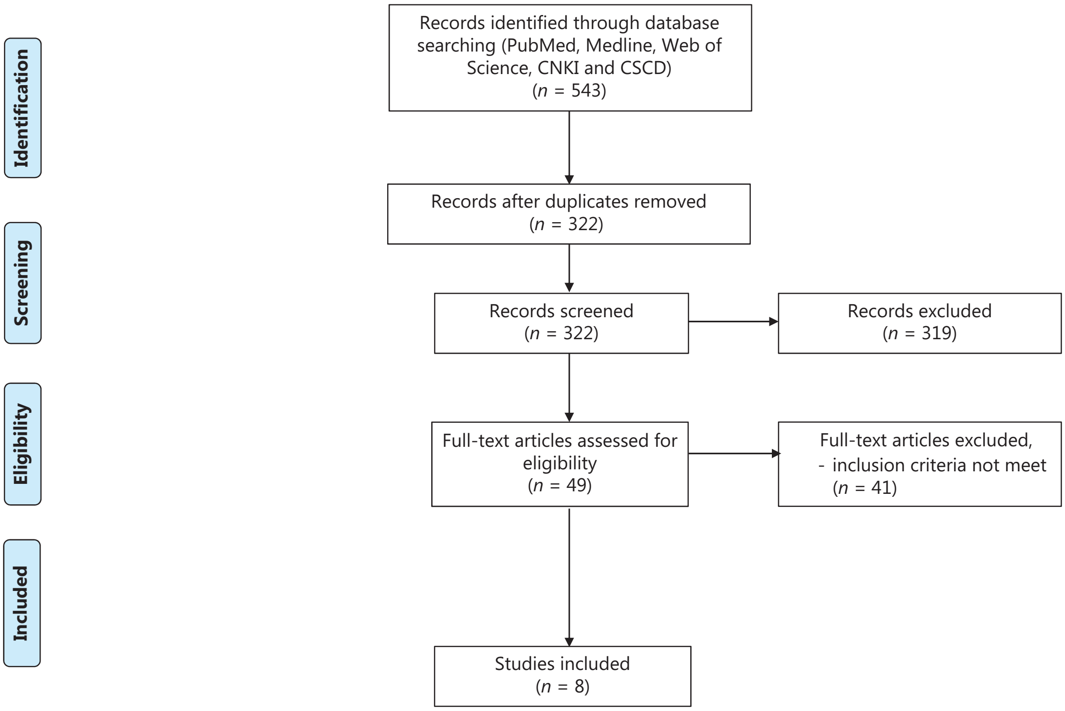

Figure S2 PRISMA flow chart of the active surveillance cohorts.
